# Efficient calculation of heterogeneous non-equilibrium statistics in coupled firing-rate models

**DOI:** 10.1186/s13408-019-0070-7

**Published:** 2019-05-09

**Authors:** Cheng Ly, Woodrow L. Shew, Andrea K. Barreiro

**Affiliations:** 1 Department of Statistical Sciences and Operations Research, Virginia Commonwealth University, Richmond, USA; 2 Department of Physics, University of Arkansas, Fayetteville, USA; 3 Department of Mathematics, Southern Methodist University, Dallas, USA

**Keywords:** Neural network model, Reduction method, Non-equilibrium statistics, Heterogeneity

## Abstract

Understanding nervous system function requires careful study of transient (non-equilibrium) neural response to rapidly changing, noisy input from the outside world. Such neural response results from dynamic interactions among multiple, heterogeneous brain regions. Realistic modeling of these large networks requires enormous computational resources, especially when high-dimensional parameter spaces are considered. By assuming quasi-steady-state activity, one can neglect the complex temporal dynamics; however, in many cases the quasi-steady-state assumption fails. Here, we develop a new reduction method for a general heterogeneous firing-rate model receiving background correlated noisy inputs that accurately handles highly non-equilibrium statistics and interactions of heterogeneous cells. Our method involves solving an efficient set of nonlinear ODEs, rather than time-consuming Monte Carlo simulations or high-dimensional PDEs, and it captures the entire set of first and second order statistics while allowing significant heterogeneity in all model parameters.

## Introduction

Advances in neural recording technologies have enabled experimentalists to simultaneously measure activity across different regions with cellular resolution [1–4]. However, it is still a technical challenge to measure the many biophysical parameters that govern this multi-region activity. This challenge is exacerbated by the fact that cortical neurons are heterogeneous (i.e., parameters vary across cells) [5] and have significant trial-to-trial noise [6]. Given these features, computational modeling of neural networks often requires exploration of a high-dimensional parameter space and lengthy, time-consuming Monte Carlo simulations. Thus, efficient methods to simulate [7] or approximate network statistics [8] are needed. Aside from computational benefits, streamlined equations for network activity offer potential benefits for mathematical analysis.

We previously developed a fast approximation method [9] for the complete first and second order statistics of a firing-rate network model based on the Wilson–Cowan model [10], and applied it to the olfactory sensory pathway [11]. However, those methods assumed that the statistics of neural activity are stationary (i.e., in steady state). Many neural systems rely on processing of time-varying, high frequency stimuli. The resulting neural responses are often transient, and a quasi-steady-state (*QSS*) approximation fails to capture the actual response statistics. For example, in the rodent vibrissa sensory [12], auditory [13–15], and electrosensory systems [16], stimuli and responses modulate on the order of a few milliseconds, i.e., much faster than the membrane time constants of neurons. Indeed, there is evidence that coding capabilities strongly depend on the timing of stimuli [17] (e.g., in the olfactory bulb [18–20]), further necessitating accurate modeling of time-varying neural activity. Modeling studies show the need to account for time-varying stimuli in calculating spiking statistics [21] and in capturing neural mechanisms such as divisive gain modulation [22]. Mathematical theory to efficiently characterize non-equilibrium heterogeneous spiking statistics is scarce despite the potential to shed light on crucial transient neural responses. Thus, it is clear that accurate modeling of time-varying neural activity would benefit mechanistic investigations of neural processing.

Here we present a method to approximate the non-equilibrium statistics of a general heterogeneous coupled firing-rate model of neural networks receiving background correlated noise, in which we: (i) assume weak coupling; equivalently, that neural activity is pairwise normal, and (ii) account for the entire probability distribution of inputs. The result is a computationally fast method because it requires the user to solve coupled nonlinear ODEs, rather than to simulate and average many realizations of coupled SDEs or numerically solve a high-dimensional PDE. The method performs much better than the related QSS method [9] in several representative examples; our code is freely available (see Availability of data and materials section).

## Model equations and method

**Figure 1:**
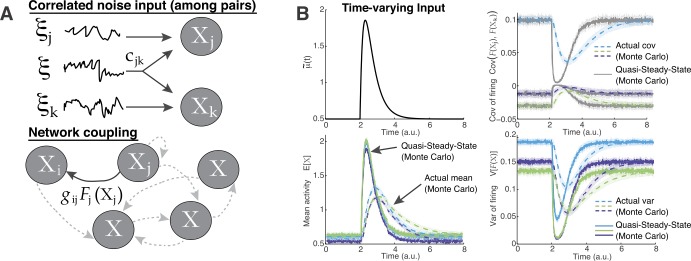
(**A**) Schematic of network model. Top: Cells receive background correlated noise $\xi_{j}(t)=\tilde{\sigma}_{j}\eta_{j}(t)$. Bottom: Network coupling via nonlinear function of activity that we choose to be a sigmoidal function. (**B**) A network of $N_{c}=3$ coupled cells with randomly chosen parameters. With fast input $\mu(t)$ (top-left) relative to the time scale, the actual non-equilibrium statistics (dash curves) are very different from the quasi-steady-state, or QSS (fixed $\mu(t)$ at time *t*, solid curves). Upper right shows all three pairs of covariance of firing $\operatorname {Cov}(\nu _{j},\nu_{k})$ for ($j\neq k$); bottom row shows the mean activity $\mathbb{E}[X_{j}]$ and variance of firing $\operatorname {Var}(\nu_{j})$. In all Monte Carlo simulations here and throughout the paper, we used 1 million realizations; see Sect. 2.3

Each cell is modeled by a single activity variable $x_{j}$, which may represent membrane voltage, calcium concentration, etc., and which evolves according to the following equation:

1 $$\begin{aligned} \tau _{j} \frac{dx_{j}}{dt} = -x_{j} + \tilde{\mu }_{j} + \tilde{\sigma }_{j} \eta _{j}(t) + \sum_{k=1}^{N_{c}} g_{jk} F_{k}\bigl(x _{k}(t)\bigr), \quad j=1,2, \dots ,N_{c} \end{aligned}$$

(see [10]), where $F_{k}(\cdot )\geq 0$ is a nonlinear function mapping input activity to firing rate or response (often called the F-I curve). All cells receive background noise $\eta _{j}$ uncorrelated in time but instantaneously correlated across different cells: $\langle \eta _{j}(t) \rangle = 0$, $\langle \eta _{j}(t) \eta _{j}(t') \rangle = \delta (t-t')$, and $\langle \eta _{j}(t) \eta _{k}(t') \rangle = c_{jk} \delta (t-t')$ for $j\neq k$ with $c_{jk}\in (-1,1)$. The parameters ${\tilde{\mu }}_{j}$ and ${\tilde{\sigma }}_{j}$ model background noisy input. The parameter $g_{jk}$ represents coupling strength from the presynaptic *k*th cell and is a signed quantity; $g_{jk}<0$ represents inhibitory coupling (Fig. 1(A)).

We wish to compute all of the first and second order time-varying statistics:

$$\begin{aligned}& \text{Mean activity} \quad \mu _{j}(t) := \langle x_{j} \rangle (t), \\& \text{Variance of activity} \quad \sigma _{j}^{2}(t) := \bigl\langle x^{2}_{j} \bigr\rangle (t) - \mu _{j}^{2}(t), \\& \text{Covariance of activity} \quad \operatorname {Cov}_{j,k}(t) := \langle x_{j} x_{k} \rangle (t) - \mu _{j}(t)\mu _{k}(t), \\& \text{Mean firing} \quad \nu _{j}(t) := \bigl\langle F_{j}(x_{j}) \bigr\rangle (t), \\& \text{Variance of firing} \quad \operatorname {Var}\bigl(\nu _{j}(t)\bigr) := \bigl\langle F^{2}_{j}(x_{j}) \bigr\rangle (t) - \nu ^{2} _{j} (t) , \\& \text{Covariance of firing} \quad \operatorname {Cov}(\nu _{j},\nu _{k};t) := \bigl\langle F_{j}(x_{j})F_{k}(x_{k}) \bigr\rangle (t) - \nu _{j}(t)\nu _{k}(t), \end{aligned}$$

where the angular brackets $\langle \cdot \rangle $ denotes averaging over realizations.

### Reduction of the Fokker–Planck equation

The corresponding probability density function $p(\vec{x},t)$ of $\vec{X}:=(x_{1},\dots ,x_{N_{c}})$, defined as $p(\vec{x},t) \,d \vec{x}=P(\vec{X(t)}\in (\vec{x}, \vec{x}+dx))$, satisfies the Fokker–Planck equation [23]:

2 $$\begin{aligned} \frac{\partial p(\vec{x},t)}{\partial t} =& -\sum_{l=1}^{N_{c}} \frac{ \partial }{\partial x_{l}} \Biggl\{ \frac{1}{\tau _{l}} \Biggl[-x_{l}+ \tilde{\mu }_{l}+\sum_{k=1}^{N_{c}} g_{lk}F_{k}(x_{k}) \Biggr] p( \vec{x},t) \Biggr\} +\frac{1}{2}\sum_{j,k} D_{j,k} \frac{\partial ^{2} p( \vec{x},t)}{\partial x_{j}\partial x_{k}} \\ = & -\sum_{l=1}^{N_{c}} \frac{\partial }{\partial x_{l}} J_{l}( \vec{x},t)+\frac{1}{2}\sum_{j,k} D_{j,k}\frac{\partial ^{2} p(\vec{x},t)}{ \partial x_{j}\partial x_{k}}, \end{aligned}$$

where $D_{j,k}=c_{jk}\frac{\tilde{\sigma }_{j} \tilde{\sigma }_{k}}{ \tau _{j}\tau _{k}}$ (see Table 1), and the sum with $D_{j,k}$ is taken over all $N_{c}\times N_{c}$ pairs of $(j,k)$. For convenience we have defined the *probability flux* or *current*, as $J_{l}(\vec{x},t) :=\frac{1}{\tau _{l}} [-x _{l}+ \tilde{\mu }_{l}+\sum_{k=1}^{N_{c}} g_{lk}F_{k}(x_{k}) ] p(\vec{x},t)$ in the right-most part of Eq. (2). This high-dimensional partial differential equation contains all of the statistics of the system.

**Table 1:** For convenience, we abbreviate the following quantities. When $j=k$ in the double integrals of $\mathcal{M_{F}}$, the bivariate normal distribution $\varrho_{j,k}$ is replaced with the standard normal distribution $\varrho_{1}$. Note that order of the arguments matters in $\mathcal{M_{F}}$: $\mathcal{M_{F}}(j,k)\neq\mathcal{M_{F}}(k,j)$ in general. The quantities in bottom three rows depend on the statistics of the activity $\mu(\cdot)$, $\sigma( \cdot)$

Abbreviation	Definition
$\varrho_{1}(y)$	$\frac{1}{\sqrt{2\pi}} e^{-y^{2}/2}$
$\varrho_{j,k}(y_{1},y_{2})$	$\frac{1}{2\pi \sqrt{1-c_{jk}^{2}}}\exp\left(-\frac{1}{2}{\vec{y}}^{T}{\left(\begin{array}{cc} 1 & c_{jk}\\ c_{jk} & 1 \end{array}\right)}^{-1}\vec{y}\right)$
$D_{j,k}$	$c_{jk}\frac{\tilde{\sigma}_{j} \tilde{\sigma }_{k}}{\tau_{j}\tau_{k}}$
$\mathcal{E}_{1}(k)$	$\int F_{k}(\sigma_{k}(t) y+\mu _{k}(t))\varrho_{1}(y)\,dy$
$\mathcal{E}_{2}(k)$	$\int F_{k}^{2}(\sigma_{k}(t) y+\mu _{k}(t))\varrho_{1}(y)\,dy$
$\mathcal{M}_{F}(j,k)$	$\iint F_{k}(\sigma_{k}(t) y_{1}+\mu_{k} (t)) y_{2} \varrho_{j,k}(y_{1},y_{2})\,dy_{1}\,dy_{2}$

### Moment closure methods

One way to tackle high-dimensional systems is through “moment closure” methods, in which state variables are integrated or averaged out, and assumptions on moments used to reduce the number of equations. Such approaches have been used in the physical [24, 25] and life sciences [26–28]; see [29] for another type of reduction method for this kind of equation. Here, we propose a closure based on weak coupling, and therefore pairwise Gaussianity in the activity variables.

Without coupling, i.e. $g_{jk}=0$, the steady-state solution of Eq. (2) is simply a multivariate Gaussian distribution with mean $\vec{\mu }=[\tilde{\mu }_{1},\dots ,\tilde{\mu }_{N_{c}}]$ and covariance matrix $\operatorname {Cov}_{j,k}=\frac{c_{jk}}{\tau _{j}+\tau _{k}} \tilde{\sigma }_{j} \tilde{\sigma }_{k}$ in the steady state. This motivates a closure of the system in which we assume *X⃗* is Gaussian: i.e. $X_{j}=\sigma _{j}+Y_{j} \mu _{j}$, where $Y_{j} $ is a standard normal random variable, with parameters $\mu _{j}$ and $\sigma _{j}$ to be determined. We also assume the joint marginal distributions are bivariate Gaussian:

3 $$P(x_{j},x_{k}):=\int p(\vec{x},t)\;d{\hat{x}}_{j,k};\;(X_{j},X_{k})∼N\left(\begin{array}{c} \left(\begin{array}{c} \mu_{j}\\ \mu_{k} \end{array}\right),\left(\begin{array}{cc} \sigma_{j}^{2} & c_{jk}\sigma_{j}\sigma_{k}\\ c_{jk}\sigma_{j}\sigma_{k} & \sigma_{k}^{2} \end{array}\right) \end{array}\right),$$

where $\mathbb{N}$ denotes a bivariate Gaussian distribution, and $d\widehat{x}_{j,k}$ denotes integrating over all $N_{c}$ variables except $x_{j}$ and $x_{k}$.

Note that the integrated quantity $\int \frac{\partial p(\vec{x},t)}{ \partial t}\,d\vec{x} = 0$, as any probability distribution must integrate to unity. We multiply Eq. (2) by $x_{j}$ and integrate the equation over all $N_{c}$ variables, $d\vec{x}=dx_{j}\,d\widehat{x}_{j}$ (where again $d\widehat{x}_{j}=dx_{1}\cdots dx_{j-1}\,dx _{j+1}\cdots dx_{N_{c}}$):

4 $$\begin{aligned}& \frac{d\mu _{j}(t)}{dt} = - \int \sum_{l=1}^{N_{c}} \frac{\partial }{ \partial x_{l}} J_{l}(\vec{x},t) x_{j}\,dx_{j} \,d\widehat{x}_{j} + \frac{1}{2} \int \sum_{l_{1},l_{2}}D_{l_{1},l_{2}} \frac{\partial ^{2} p(\vec{x},t)}{\partial x_{l_{1}}\partial x_{l_{2}}} x_{j}\,dx_{j}\,d\widehat{x}_{j}, \end{aligned}$$

where $\frac{d\mu _{j}(t)}{dt} = \frac{\partial }{\partial t} \int x _{j} p(\vec{x},t)\,d\vec{x}$. Consider the first term on the RHS: when $l\neq j$, we have $\int \frac{\partial }{\partial x_{l}} J_{l}( \vec{x},t) x_{j}\,dx_{j}\,d\widehat{x}_{j}= \int \frac{\partial }{ \partial x_{l}} J_{l}(\vec{x},t)\,dx_{l} x_{j}\,dx_{j}\,d\widehat{x}_{l, j} =\int J_{l}\vert _{x_{l}=-\infty }^{x_{l}=\infty } x _{j}\,dx_{j}\,d\widehat{x}_{j} = \int 0 x_{j}\,dx_{j}\,d\widehat{x}_{j} = 0$. The last equality comes from no flux at ±∞: $J_{l} \vert _{x_{l}=-\infty }^{x_{l}=\infty }=0$. A similar calculation applies to the second term, for all $N_{c}\times N_{c}$ values of $(l_{1},l_{2})$: when $l_{1}\neq j$ and $l_{2}\neq j$, first integrate in $x_{l_{1}}$ and $x_{l_{2}}$, and then use the fact that there is no density at ±∞: $p(\vec{x},t) \vert _{x_{l_{1/2}}=-\infty }^{x_{l_{1/2}}=\infty }=0$; when $l_{1/2}=j$, first integrate in $x_{j}$, then integrate by parts, using $\partial _{j} p(\vec{x},t) x_{j}\vert _{x_{j}=-\infty }^{x_{j}=\infty }=0$ and $\partial _{j} p(\vec{x},t) \vert _{x_{j}=-\infty }^{x_{j}= \infty }=0$. Therefore, Eq. (4) becomes

5 $$ \frac{d\mu _{j}(t)}{dt} =\frac{1}{\tau _{j}} \Biggl( -\mu _{j}(t) + \tilde{ \mu }_{j} + \sum_{k=1}^{N_{c}} g_{jk} \mathcal{E}_{1}(k) \Biggr) , $$

where we have used the approximation $\int F_{k}(x_{k})p(\vec{x},t)\,d\vec{x}\approx \mathcal{E}_{1}(k)$ (see Table 1) by assuming the marginal $x_{k}$ PDF is a normal distribution with mean $\mu _{k}(t)$ and variance $\sigma _{k}^{2}(t)$.

To derive a similar equation for the variance $\sigma _{j}^{2}(t)$, we multiply Eq. (2) by $x_{j}^{2}$ and again integrate over all variables:

6 $$\begin{aligned}& \frac{d E_{j^{2}}(t)}{dt} = - \int \sum_{l=1}^{N_{c}} \frac{\partial }{\partial x_{l}} J_{l}(\vec{x},t) x_{j}^{2} \,dx_{j}\,d\widehat{x} _{j} + \frac{1}{2} \int \sum_{l_{1},l_{2}}D_{l_{1},l_{2}} \frac{ \partial ^{2} p(\vec{x},t)}{\partial x_{l_{1}}\partial x_{l_{2}}} x _{j}^{2}\,dx_{j}\,d \widehat{x}_{j} , \end{aligned}$$

where $E_{j^{2}}(t)=\int x^{2}_{j} p(\vec{x},t)\,d\vec{x}$, and $\sigma ^{2}_{j}(t)=E_{j^{2}}(t)- (\mu _{j}(t) )^{2}$.

Following the same type of manipulations and again using the no density condition at ±∞: $p(\vec{x},t)\vert _{x_{l_{1/2}}=-\infty } ^{x_{l_{1/2}}=\infty }=0$, we get

7 $$ \frac{d E_{j^{2}}(t)}{dt} =D_{j,j}+ \frac{2}{\tau _{j}} \Biggl[ -E_{j ^{2}}(t) + \tilde{\mu }_{j} \mu _{j}(t)+ \sum _{k=1}^{N_{c}} g_{jk} \int x_{j} F_{k}(x_{k}) p(\vec{x},t)\,d \vec{x} \Biggr] . $$

We now employ our approximation, $x_{j}=\mu _{j}(t)+y_{j}\sigma _{j}(t)$ where $y_{j}$ is a standard normal random variable, to close the last term in Eq. (7). We further approximate $\int y _{j} F_{k}(\mu _{k}(t)+y_{k}\sigma _{k}(t))p(\vec{x},t)\,d\vec{x}$ by assuming the joint marginal distribution of $(x_{j},x_{k})$ is bivariate normal, and use the definition of $\mathcal{M_{F}}$ in Table 1: $\int y_{j} F_{k}(\mu _{k}(t)+y_{k} \sigma _{k}(t))p(\vec{x},t)\,d\vec{x} \approx \mathcal{M_{F}}(j,k)$. Therefore, the equation for the second moment is

8 $$ \tau _{j} \frac{d E_{j^{2}}(t)}{dt} = \frac{\tilde{\sigma }^{2}_{j} }{ \tau _{j}} + 2 \Biggl[ -E_{j^{2}}(t)+ \tilde{\mu }_{j} \mu _{j}(t) + \sum_{k=1}^{N_{c}} g_{jk} \bigl( \mu _{j}(t)\mathcal{E} _{1}(k)+\sigma _{j}(t) \mathcal{M_{F}}(j,k) \bigr) \Biggr] . $$

To derive the analogous equation for the $\operatorname {Cov}_{j,k}(t)$, the procedure is almost exactly the same except that Eq. (2) is multiplied by $x_{j} x_{k}$, and two terms from the sum (over probability fluxes $J_{l}$) contribute, when $l=j$ and $l=k$. The result is

9 $$\begin{aligned} \tau _{j}\tau _{k} \frac{d E_{j,k}(t)}{dt} = & c_{jk} \tilde{\sigma } _{j}\tilde{\sigma }_{k} + \tau _{k} \biggl( - E_{j,k}+\tilde{\mu }_{j}\mu _{k}(t)+\sum_{l} g_{jl} \bigl( \mu _{k}(t)\mathcal{E}_{1}(l)+\sigma _{k}(t) \mathcal{M_{F}}(k,l) \bigr) \biggr) \\ & {}+ \tau _{j} \biggl( - E_{j,k}+\tilde{\mu }_{k}\mu _{j}(t)+\sum_{l} g _{kl} \bigl( \mu _{j}(t)\mathcal{E}_{1}(l)+\sigma _{j}(t) \mathcal{M_{F}}(j,l) \bigr) \biggr) . \end{aligned}$$

When $j=k$ in Eq. (9), we recover Eq. (8).

The full set of kinetic equations given by Eq. (5), (8), and (9) form a system of nonlinear coupled ODEs with $N_{c} + N_{c}(N_{c}+1)/2$ variables. The statistics of the firing rate (i.e. $\nu _{j} = F_{j}(x_{j})$) are obtained from a standard change of variables.

If *μ̃*, *σ̃* are constant in time, the system (Eq. (5), (8), (9)) settles to a steady state:

10 $$\begin{aligned}& \begin{aligned} & \mu _{j} = \tilde{\mu }_{j} + \sum_{k=1}^{N_{c}} g_{jk} \mathcal{E}_{1}(k),\qquad \sigma _{j}^{2} = \frac{\tilde{\sigma }^{2}_{j}}{2\tau _{j}} +\sigma _{j} \sum_{k=1}^{N_{c}} g_{jk} \mathcal{M_{F}}(j,k), \\ & \operatorname {Cov}_{j,k}\frac{\tau _{j}+\tau _{k}}{2} = c_{jk}\frac{ \tilde{\sigma }_{j} \tilde{\sigma }_{k}}{2} + \frac{\sigma _{j}(t)}{2} \tau _{j} \sum_{l=1}^{N_{c}} g_{kl} \mathcal{M_{F}}(j,l) + \frac{\sigma _{k}(t)}{2} \tau _{k} \sum_{l=1}^{N_{c}} g_{jl} \mathcal{M_{F}}(k,l). \end{aligned} \end{aligned}$$

A common approximation to non-equilibrium statistics is to assume that the system immediately equilibrates to the steady-state solution of Eq. (1) at each time point for the time-dependent parameters $\tilde{\mu }_{j}(t)$, $\tilde{\sigma }_{j}(t)$, which we call the QSS method. We will find that the QSS method fails to capture meaningful features of network activity with relatively fast input.

### Monte Carlo simulations

In the Results section, we compare our new method with Monte Carlo simulations. For all Monte Carlo simulations (i.e., both the actual non-equilibrium statistics and QSS), we used 1 million ($1\times 10^{6}$) realizations at *each* time point. The shaded error regions in all figures represent 1 standard deviation above and below the mean, which is approximated via the sample standard deviation on 1000 samples of 1000 realizations each: $S=\sqrt{\frac{1}{999} \sum_{j=1}^{1000} ( X(j)- \overline{X} ) ^{2} }$, where *X̅* is the average over 1 million realizations and $X(j)$ is an average over 1000 realizations.

## Results

**Figure 2:**
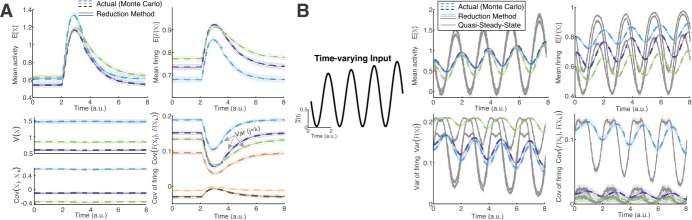
(**A**) Our method (solid) is very accurate in capturing the results of Monte Carlo (dashed) (cf. Fig. 1(B)) for all first and second order non-equilibrium statistics. (**B**) With fast sinusoidal input $\mu(t)$ (left), the actual non-equilibrium statistics (dashed) are very different from QSS (gray curves), but again our method accurately captures the statistics

**Figure 3:**
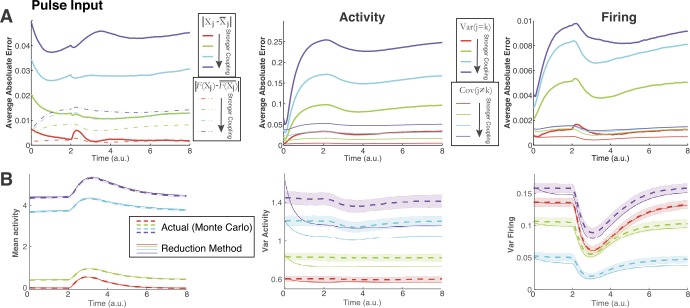
Applying our method to a larger network of $N_{c}=50$ neurons. As coupling strength increases (red → green → cyan → purple), performance worsens. (**A**) The absolute value of the error of our method with the Monte Carlo simulations as a function of time. Each *Average Absolute Error* time point is averaged over the entire set of statistics (i.e., for the mean and variances the average is over all 50, for covariances the average is over $1225=49*25$). (**A**) Left: the average for the mean activity $X_{j}$ (solid) and mean firing $F(X_{j})=\nu_{j}$ (dot-dashed); with the progression of colors (red to purple) representing stronger (i.e., larger) coupling values $G_{j,k}$. (**A**) Middle: Error of covariances (thinner lines, $j\neq k$) and variances (thicker lines, $j=k$) of activity $X_{j}$. (**A**) Right: Error of covariances (thinner lines, $j\neq k$) and variances (thicker lines, $j=k$) of firing $F(X_{j})=\nu_{j}$. (**B**) Representative comparisons of our method with the Monte Carlo simulations. (**B**) Left: although the average error increases with coupling magnitude, the discrepancies are not noticeable for mean activity and firing (not shown). (**B**) Middle: the method is visibly worse for the variance of activity as coupling magnitude increases. (**B**) Right: the method is visibly worse as coupling magnitude increases – note that the weakest coupling (red) is between green (second weakest) and purple (strongest)

**Figure 4:**
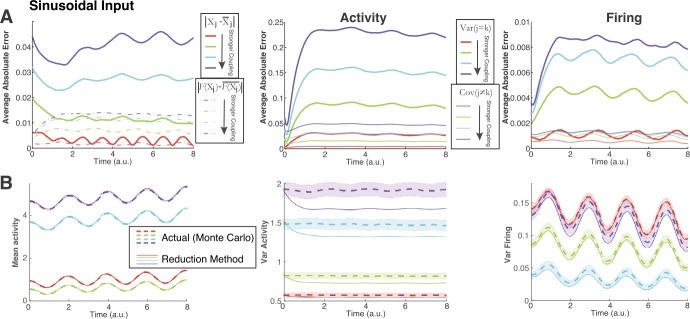
Applying our method to a larger network of $N_{c}=50$ neurons. Same format as Fig. 3 except with sinusoidal input (see Fig. 2(B)). (**A**) Again as coupling strength increases (red → green → cyan → purple), performance worsens. (**B**) Representative comparisons of our method with the Monte Carlo simulations. (**B**) Left: although the average error increases with coupling magnitude, the discrepancies are not noticeable for mean activity and firing (not shown). (**B**) Middle: the method is visibly worse for the variance of activity as coupling magnitude increases. (**B**) Right: the method is visibly worse as coupling magnitude increases for variance of firing – note that the weakest coupling (red) has largest variance of firing

We implement our method for networks of various sizes $N_{c}$, with two time-varying inputs. We choose *F* to be a sigmoidal: $F_{j}(\cdot ) = 0.5(1+\tanh ((x-x_{\mathrm{rev},j})/x_{\mathrm{sp},j}))\in [0,1]$ (arbitrary units, $x_{\mathrm{rev},j}$ and $x_{\mathrm{sp},j}$ are parameters). To include heterogeneity, parameters were chosen randomly from the following distributions:

11 $$ \begin{aligned} &\tau _{j} \sim \mathbb{N} \bigl(1,0.1^{2}\bigr), \qquad {\tilde{\mu }}_{j} \sim \mathbb{U}-0.5, \qquad {\tilde{\sigma }}_{j} \sim \mathbb{U}+1, \\ &x_{\mathrm{rev},j} \sim \mathbb{N}\bigl(0,0.1^{2}\bigr), \qquad x_{\mathrm{sp},j} \sim 0.35\mathbb{U}+0.05, \end{aligned} $$

where $\mathbb{U}\in [0,1]$ is a uniform random variable, and $\mathbb{N}$ is a normal random variable. The input correlation matrix **Cr** was generated so to have approximately independent off-diagonal entries as follows: (i) create a matrix **A** with i.i.d. entries $\mathbf{A}_{jk}\sim \mathbb{N}(0,0.8^{2})$; (ii) create a diagonal matrix $\boldsymbol{\varLambda }_{\vec{d_{s}}}$ from the vector $\vec{d_{s}}$ where $\vec{d}_{s}(j)=1/ \sqrt{(\mathbf{A}^{T}{\mathbf{A}})_{jj}}$; (iii) set $\mathbf{Cr} = ( \boldsymbol{\varLambda }_{\vec{d_{s}}}) \mathbf{A}^{T}{\mathbf{A}} ( \boldsymbol{\varLambda }_{\vec{d_{s}}})$. By construction, **Cr** is symmetric positive semidefinite with 1’s on the diagonal. Finally, the entries of the coupling matrix **G** are independently chosen: $\mathbf{G}_{jk} \sim \mathbb{N}(0,v_{l})$ where $v_{l}=(l/10)^{2}$ with $l=1$ for Figs. 1–2, and $l=1,2,3$, or 4 in Figs. 3–4. All entries of **G** are nonzero (i.e. coupling is all-to-all), with inhibitory, excitatory, and self-coupling cases.

Figure 1(B) shows that with relatively fast time-varying $\mu (t)$, a network of $N_{c}=3$ cells has complex non-equilibrium network statistics that cannot be approximated by the QSS approximation (i.e., assuming the system immediately equilibrates to the steady-state solution for each time point). This is true for the complete set of activity and response statistics, although for brevity only a subset are shown. All parameters are chosen as in Eq. (11) except for $\mu (t)$, which is the same for all three cells.

Figure 2(A) shows that the time-varying method (Eq. (5), (8), and (9)), when applied to same network as in Fig. 1(B), gives accurate results for the complete set of first/second order statistics. Figure 2(B) shows a detailed comparison of another instance of the $N_{c}=3$ cell network, but with a time-varying sinusoidal input. Again, the QSS method does not capture the actual network statistics, but our method does very well (colored solid curves). We only show a subset of statistics to illustrate our point; the others are qualitatively similar.

Thus far we have only consider small networks. In Figs. 3 and 4, our methods are applied to a large network of $N_{c}=50$ coupled cells where the magnitude of the coupling strengths vary: $\mathbf{G}_{jk}\sim \mathbb{N}(0,\frac{l^{2}}{100})$, for $l=1,2,3,4$. Figure 3 shows the results with pulse input (Fig. 1(B) upper-left) applied to all 50 cells, while Fig. 4 shows results from applying the sinusoidal input (Fig. 2(B) left) to all 50 cells. Figure 3(A) (top row) shows the error between our method and the actual (Monte Carlo) statistics; we plot the absolute error averaged over all cells or pairs:

$$ \text{Average Absolute Error} = \frac{1}{M} \sum _{j=1}^{M} \bigl\vert X_{\text{MC}}(t) - X_{\text{Method}}(t) \bigr\vert , $$

i.e., for mean and variance of activity and firing, $M=50$; for covariance of activity and firing, averaging over all $M=50*49/2$ distinct pairs. All six sets of statistics are shown in Fig. 3(A): the left panel shows the average absolute error for both mean activity (solid) and mean firing (dot-dashed), middle panel shows the variance (thick solid) and covariance of activity (thin solid), the right panel shows the variance (thick solid) and covariance (thin solid) of firing. In all cases, as the coupling magnitude increases (red → green → cyan → purple), the error increases. For reference, the bottom row (Fig. 3(B)) compares our method with the Monte Carlo simulations for a particular cell (or cell pair); the chosen cell or pair is the one that most closely matches the average absolute error. In Fig. 3(B), we only show three out of the six statistics (left is mean activity, middle is variance of activity, right is variance of firing) because these clearly show the performance of our method in relation to the size of the average absolute error. Figure 4 has exactly the same format as Fig. 3, but with sinusoidal input.

**Figure 5:**
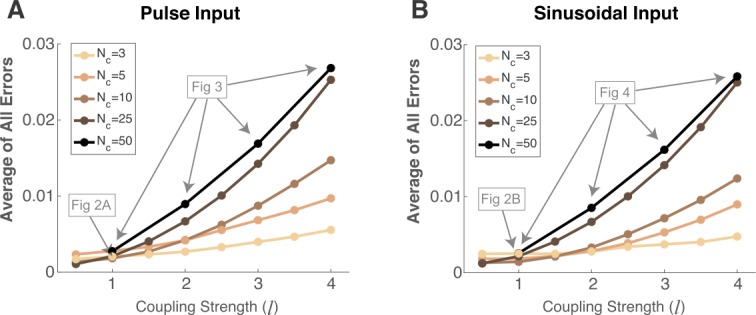
Our method implicitly assumes weak coupling, so as the average magnitude of the coupling strength increases, the performance decreases. We demonstrate this with several instances of coupling matrices and network sizes $N_{c}=3, 5, 10, 25$, and $N_{c}=50$ with the four coupling values in Figs. 3 and 4, using the same pulse (**A**) and sinusoidal (**B**) inputs. On vertical axis, we plot the average absolute error over *all* first and second order statistics, including all cells and pairs, while on the horizontal axis, we plot a measure of average magnitude of the coupling values *l*. Note that, since $\mathbf{G}_{jk}\sim\mathbb{N}(0,\frac{l ^{2}}{100})$, the average of all $\vert{\mathbf{G}}_{jk} \vert$ is $\frac{l}{5\sqrt{2\pi}}$ in the infinite limit $N_{c}\to\infty$. For reference, some of the points on these curves are from prior figures, denoted in gray text and arrows

Finally, in Fig. 5, to assess the performance of our method, we plot the absolute value of the error averaged over all six statistics and over all cells/pairs (vertical axis) as a function of a measure of coupling strength *l* (Fig. 5(A) is with pulse input, (B) with sinusoidal input). Each curve shows a different network size, ranging from $N_{c}=3, 5, 10, 25, 50$, with a particular instance of randomly chosen parameters for each curve.^1^ The magnitude of the coupling strength, *l*, on the horizontal axis is from $\mathbf{G}_{jk}\sim \mathbb{N}(0,\frac{l^{2}}{100})$, so that the average of all $N_{c}^{2}$ values of $\vert {\mathbf{G}}_{jk} \vert $ is $\frac{l}{5\sqrt{2\pi }}$ in the infinite limit $N_{c}\to \infty $. Not surprisingly, the average error increases as coupling strength increases for each curve. Assessing how much absolute error is acceptable depends on the purposes of the approximation, but for reference, the instances of networks from prior figures are denoted in gray. Figure 5 indicates that, as long as the average absolute error is below 0.01, our method likely performs very well, independent of network size (cf. with Figs. 2–4). Average absolute errors larger than 0.01 might indicate at least some of the statistics calculated by our method are likely to be inaccurate, although others may be accurate depending on cell or pair (cf. Figs. 3–4).

## Conclusion

The role of mathematical theory and computation in addressing neuroscience questions is as vital as ever despite tremendous advances in recording technologies. As detailed in the Introduction, the common assumption of equilibrium neural network responses is inaccurate in many neural systems. Here we derived and implemented a reduction method to calculate the complete set of first and second order *non-equilibrium* statistics in coupled heterogeneous networks of firing-rate models [10] receiving background correlated noise [30–32]. Importantly, our method captures the non-equilibrium statistics when they are vastly different from the quasi-steady-state, and works very well even with significant heterogeneity in all model parameters. As the overall magnitude of the coupling strengths increase, the performance of our method declines because the moment closure method assumes weak coupling.

Mathematical reductions that well approximate the statistics of firing-rate models [33], such as the one described here, are likely to be relevant for future theoretical studies of neural networks for several reasons. Wilson–Cowan type models [10] are commonly used because of their simplicity and history of successful application in neural systems. Analysis of spiking statistics using mean-field methods often results in similar firing-rate equations [34–37]. Finally, such methods might be useful for mechanistic investigations of neural function across multiple brain regions that commonly rely on larger models with more parameters and complexity [7, 11].

## Footnotes

1 On each curve, the intrinsic parameters are randomly chosen and fixed as *l* varies. The same realization of $\mathbf{G}_{jk}$ is used for each curve, and simply scaled by *l* to vary the coupling strength.

## Abbreviations

QSS: Quasi-Steady-State
RHS: Right-hand side
ODEs: Ordinary differential equations
PDEs: Partial differential equations
SDEs: Stochastic differential equations
